# Cysticercose disséminée à localisation neurologique, oculaire et cutanée chez une patiente sénégalaise

**DOI:** 10.11604/pamj.2020.36.379.22722

**Published:** 2020-08-31

**Authors:** Bruce Shinga Wembulua, Kalilou Diallo, Moussa Diallo, Marie Antoinette Daba Dione, Abdoulaye Diop, Noel Magloire Manga

**Affiliations:** 1 Service des Maladies Infectieuses et Tropicales, Centre Hospitalier National Universitaire de Fann, Dakar, Sénégal,; 2 Unité des Maladies Infectieuses et Tropicales, Université Assane Seck, Hôpital de la Paix, Ziguinchor, Sénégal,; 3 Service de Dermatologie et Vénéréologie, Centre Hospitalier National Universitaire Aristide Le Dantec, Dakar, Sénégal,; 4 Unité de Dermatologie et Vénéréologie, Université Assane Seck, hôpital de la Paix, Ziguinchor, Sénégal,; 5 Unité de Neurochirurgie, Université Assane Seck, hôpital régional, Ziguinchor, Sénégal

**Keywords:** Cysticercose disséminée, neurocysticercose, oculaire, cutanée, Disseminated cysticercosis, brain, eye, skin

## Abstract

La cysticercose est une maladie tropicale négligée prioritaire pour l´Organisation Mondiale de la Santé. La plupart des cas rapportés sont des formes cutanées, oculaires ou neurologiques isolées. Les formes disséminées restent cependant rares. Nous rapportons un cas de cysticercose disséminée à localisation neurologique, oculaire et cutanée chez une patiente sénégalaise de 66 ans reçue pour des céphalées et des crises convulsives chroniques et chez qui l´examen clinique avait objectivé un syndrome cérébelleux associé à des lésions nodulaires sous-cutanées généralisées et indolores. Le diagnostic a été confirmé par la mise en évidence des cysticerques à l´examen histopathologique de la pièce biopsique cutanée. La patiente avait bien évolué sous Albendazole.

## Abstract (en)

Cysticercosis is a neglected tropical disease set as health priority by WHO. Most of the reported cases included isolated types of cysticercosis affecting the skin, the eyes or the brain . Disseminated types, however, are rare. We here report a case of disseminated cysticercosis affecting the brain, the eyes and the skin in a Senegalese female patient aged 66 years admitted with headaches and chronic seizures. Clinical examination showed cerebellar syndrome associated with generalized and painless nodular subcutaneous lesions. Diagnosis was confirmed based on histopathological examination of skin biopsy which showed cysticerci. Patient’s outcome was good under albendazole therapy.

## Introduction

La cysticercose est une cestodose larvaire due à *Cysticercus cellulosae*, forme larvaire d´un ténia des porcs appelée *Taenia solium*. Elle atteint principalement les muscles, les yeux, la peau et le système nerveux [1]. En zone d´endémie notamment en Afrique subsaharienne, où de nombreux cas d´épilepsie sont liés à la neurocysticercose, la maladie reste paradoxalement méconnue [1,2]. La plupart de cas rapportés sont des formes cutanées, oculaires ou neurologiques isolées [3,4]. Les formes disséminées restent cependant rares avec moins de 150 cas rapportés dans le monde, surtout en Asie selon la revue de Qavi *et al* en 2016 [5]. Nous rapportons une observation de cysticercose disséminée à localisation neurologique, oculaire et cutanée chez une patiente sénégalaise de 66 ans.

## Patient et observation

Une patiente âgée de 66 ans était reçue au service de médecine de l´hôpital de la Paix de Ziguinchor au Sénégal pour des céphalées et des crises convulsives. Le début de la symptomatologie remontait à 6 mois marqué par des céphalées en casque d´installation progressive, d´allure intermittente à prédominance nocturne et d´intensité modérée. Ces céphalées étaient accompagnées 3 semaines plus tard des crises convulsives généralisées, sans morsure de la langue ni perte d´urine et suivies d´une brève amnésie post- critique. Les crises duraient environ 5 minutes avec une fréquence de 2 à 3 épisodes par mois. Ce tableau clinique était précédé deux ans plus tôt de lésions nodulaires sous-cutanées indolores et généralisées ayant motivé une consultation en dermatologie sans succès. Dans ses antécédents on retrouvait une notion de consommation régulière de viande de porc.

**Figure 1:**
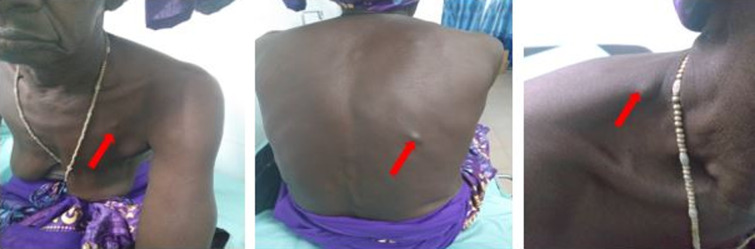
lésions nodulaires sous-cutanées disséminées de taille variée chez une patiente de 66 ans

**Figure 2:**
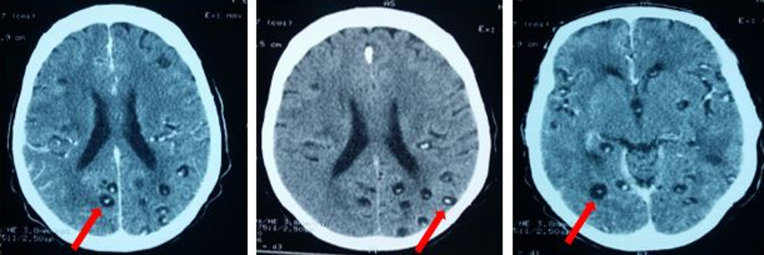
TDM cérébrale (coupe axiale) montrant des formations kystiques infra centimétriques à contenue nodulaire mural disséminées dans tout le parenchyme cérébral

**Figure 3:**
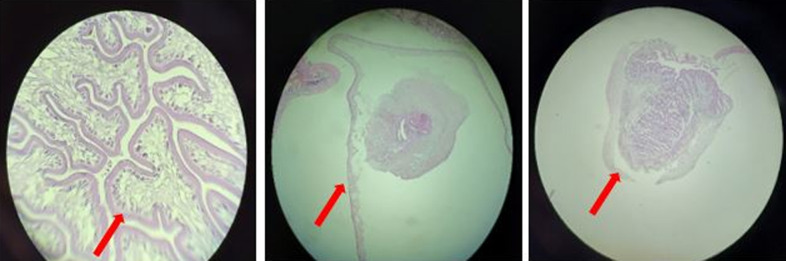
coupes histologiques montrant des formations pseudo-kystiques à paroi fibreuse épaisse, infiltrées d´éléments inflammatoires

L´examen neurologique retrouvait une conscience claire, un syndrome cérébelleux fait de trouble de l´équilibre avec signe de Romberg positif et une dysmétrie modérée à l´épreuve doigt-nez. La sensibilité et la motricité étaient conservées. L´examen dermatologique montrait des nodules sous-cutanés d´environ 1 cm localisés sur le tronc, les membres et le cuir chevelu. Ces nodules étaient durs, indolores, mobiles par rapport aux deux plans et sans fistulisation avec une peau en regard normale. (Figure 1). L´hémogramme objectivait une légère anémie à 11,5 g/dl avec les globules blancs à 5100 cell/mm^3^et une éosinophilie à 500 cell/mm^3^. La fonction rénale et l´ionogramme sanguin étaient normaux. La glycémie à jeun était à 0,81 g/l et la sérologie VIH négative. La sérologie n´avait pas été réalisée du fait de notre plateau technique limité. L´examen des selles n´avait pas objectivé de parasites. La tomodensitométrie (TDM) cérébrale avait mise en évidence des multiples formations kystiques infra-centimétriques à contenue nodulaire mural, disséminées dans tout le parenchyme cérébral (Figure 2). Le fond d´œil avait objectivé des nodules disséminés au pôle postérieur de la rétine sans signe d´hypertension intracrânienne. L´examen histopathologique de la pièce biopsique cutanée avait objectivé une formation pseudo kystique à paroi fibreuse épaisse, infiltrée d´éléments inflammatoires (lymphoplasmocytes et quelques macrophages) et dont le contenu était constitué du scolex typique d´un cysticerque (Figure 3). Le diagnostic de cysticercose disséminée à localisation neurologique, oculaire et cutanée était retenu.

La patiente a été mise sous Albendazole 15 mg/Kg pendant 28 jours, Prednisolone 1 mg/Kg/jr le matin pendant 5 jours et Phénobarbital 100 mg/jr. L´évolution après 7 jours de traitement était favorable marquée par une régression des céphalées et du syndrome cérébelleux ainsi qu´une rémission des crises convulsives. Un mois après son hospitalisation, la patiente ne présentait aucune plainte et on notait une nette régression des nodules sous-cutanées.

## Discussion

La cysticercose fait partie des 17 maladies tropicales négligées prioritaires pour l´Organisation Mondiale de la Santé (OMS) [1]. La forme disséminée relativement fréquente en Asie, reste rare et peu rapportée en Afrique [2,5]. Notre observation est, en notre connaissance, la première forme disséminée rapportée au Sénégal et en Afrique de l´ouest [2]. Le tableau clinique de cysticercose disséminée est polymorphe. Des lésions nodulaires indolores telles que retrouvées chez notre patiente sont classiques dans les atteintes cutanées et musculaires [4]. Les formes cérébrales symptomatiques se traduisent dans 80% de cas par des crises convulsives épileptiformes [6]. Le trouble de l´équilibre, rarement rapporté [5,6], fait la particularité de l´atteinte neurologique dans notre observation. Les manifestations oculaires quant à elles, varient d´une simple baisse de l´acuité visuelle à une cécité complète [7]. Des atteintes oculaires asymptomatiques comme chez notre patiente sont également décrites [8]. L´intérêt d´un fond d´œil systématique doit être discuté devant ces cas.

Le diagnostic de certitude reste histologique en montrant un aspect de pseudo-kystique à paroi épaisse contenant un scolex [5,7,8]. Une image kystique ou nodulaire calcifiée à la TDM ou IRM cérébrale dans un contexte épidémiologique évocateur, est très suggestive d´une atteinte neurologique [5,6]. L´examen des selles à la recherche des parasites n´est contributif que dans 25% de cas [2]. Lorsqu´elle est disponible, la sérologie au Western Blot est d´un grand t´intérêt surtout pour des localisations difficiles d´accès à la biopsie [9]. La spécificité et la sensibilité des tests sérologiques sont d´autant meilleures que les lésions sont multiples et actives (non calcifiées) [7]. Chez notre patiente, le diagnostic de certitude a été posée à l´histologie des pièces biopsiques cutanées alors que l´examen des selles n´avait pas permis d´isoler le parasite. L´Albendazole et/ou le Praziquantel constitue le traitement de choix de la cysticercose [10]. Il n´y a aucun consensus sur la durée du traitement des formes disséminées. Toutefois, l´Albendazole à la dose de 15 mg/kg pendant 28 jours est la conduite thérapeutique la plus utilisée dans la littérature avec des bons résultats [5]. Une courte corticothérapie est recommandée dans les atteintes neurologiques pour prévenir les complications liées à l´œdème cérébral induit par la lyse des kystes sous traitement [9,10]. Le Praziquantel étant contre indiqué dans les localisations oculaires [9], notre patiente a été mise sous Albendazole associé à une courte corticothérapie et l´évolution était favorable.

## Conclusion

La cysticercose est une maladie tropicale négligée. Les formes disséminées sont rares et cliniquement polymorphes. Habituellement bénigne, elles peuvent cependant, être de mauvais pronostic dans certaines localisations. En zone d´endémie, une localisation doit amener à rechercher d´autres.
